# A Preliminary Report Showing Spinosad and Fluralaner Are Able to Incapacitate Cimex lectularius L., the Common Bed Bug

**DOI:** 10.7759/cureus.7529

**Published:** 2020-04-03

**Authors:** Johnathan M Sheele

**Affiliations:** 1 Emergency Medicine, Mayo Clinic, Jacksonville, USA

**Keywords:** bed bug, cimex lectularius, fluralaner, spinosad, bedbug, drug, treatment, veterinary, xenointoxication

## Abstract

*Cimex lectularius* L., the common bed bug, is a hematophagous human ectoparasite. The veterinary drugs, spinosad and fluralaner, were studied for their ability to incapacitate *C. lectularius* when administered in a blood meal using an artificial feeding system under laboratory conditions. Tested drug doses were based on the reported peak blood levels in animals given the drugs. Spinosad at doses 1,000 ng/mL or higher resulted in 75% or greater bed bug incapacitation (defined as death or immobility). Fluralaner at doses 500 ng/mL or higher had 100% bed bug incapacitation. Both drugs were significantly more effective than controls at these doses (*P *< 0.001).

## Introduction

*Cimex lectularius* L., known as the common bed bug, is an obligate hematophagous insect that primarily feeds on humans [1]. The number of bed bug infestations has risen in many parts of the world in the past few decades, which is in part due to growing resistance to the pesticides used in their control [1]. Elimination of bed bug infestations relies on an integrated pest management (IPM) approach that includes decreasing insect harborage and the use of pesticides or heat treatments [1].

Under natural conditions, bed bugs take blood meals every few days [1]. Eggs laid by female *C. lectularius* hatch after about 1 week, with the first instars then seeking a blood meal [1]. Bed bugs develop through five instars before becoming sexually active adults [1].

Bed bugs are likely one of the most common human ectoparasites encountered by health care providers in industrialized countries [2-9]. No orally administered pharmaceutical agents for humans have a US Food and Drug Administration-approved indication for bed bugs. The antiparasitic drugs, ivermectin and moxidectin, both cause toxicity to bed bugs [10-15]. Ivermectin could be considered an adjective therapy along with IPM to eliminate a bed bug infestation. Moxidectin has more favorable pharmacokinetics than ivermectin, but the drug does not appear to cause as much long-lasting harm to bed bugs as ivermectin [11,16].

The veterinary drugs, spinosad and fluralaner, are currently used to control ectoparasites in animals but have not been studied for activity against bed bugs when administered in a blood meal. Spinosad, which consists of spinosyn A and D, is used to kill fleas in dogs and cats by acting at the nicotinic acetylcholine receptors in the insect nervous system [17]. Ingestion of 30 mg/kg of spinosad by dogs resulted in a peak plasma concentration of 1,550 ng/mL about 3 hours after ingestion, with a mean elimination half-life of 271 hours [18]. Spinosad has low mammalian toxicity, with an oral median lethal dose of 3,738 mg/kg for male rats and greater than 5,000 mg/kg for female rats and mice [17]. Topically applied spinosad has been shown to be toxic to bed bugs [19].

Fluralaner is part of the isoxazoline class of ectoparasiticides that inhibits the glutamate and gamma-aminobutyric acid ligand-gated chloride channels [20]. Fluralaner has high plasma protein binding and volume of distribution, which allows for therapeutic concentrations in animals for up to 12 weeks [20]. A single dose of afoxolaner, a drug in the same class as fluralaner, was 100% efficacious at clearing *Sarcoptes scabiei* (scabies) in a porcine model of human scabies [21]. In dogs, the peak plasma concentration after ingesting 25 mg/kg fluralaner varied by whether the animal was fasting (1,591 ng/mL) or fed (3,377 ng/mL) [22]. In animals, the time to peak concentration was approximately 1 day, with mean plasma concentrations greater than 500 ng/mL persisting for more than 21 days after drug dosing [22]. The aim of this study was to determine if blood meals containing fluralaner and spinosad would incapacitate *C. lectularius*.

## Materials and methods

**Figure 1:**
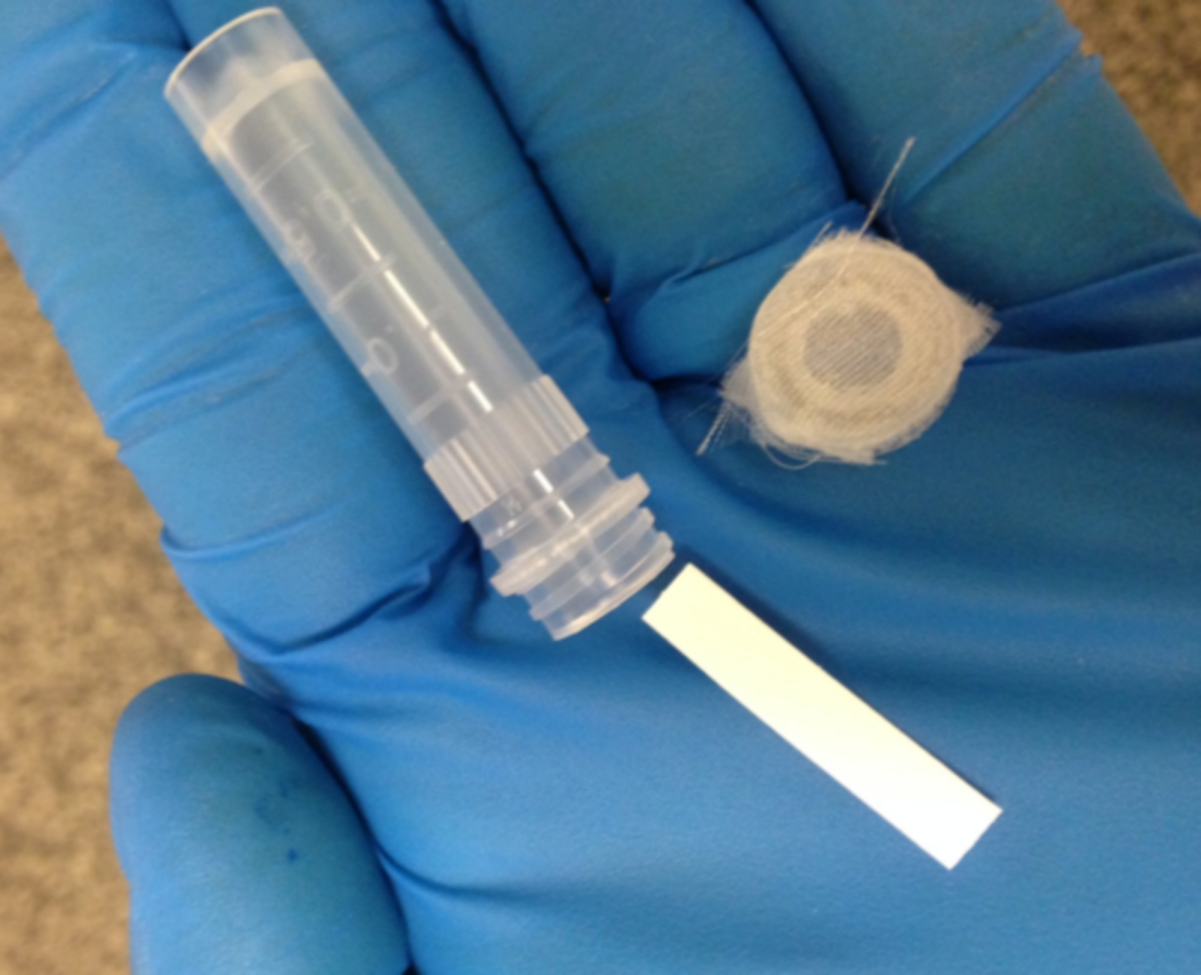
An example of a test tube that would contain the bed bugs

Mixed inbreeding populations of the Fort Dix-Harlan strain and Ridge strain of *C. lectularius* L. were used in the experiments. The numbers of nymphs and adult bed bugs were not held constant, to mimic a natural population. Bed bugs were prepared and fed as described previously [11,12,15]. Bed bugs were kept in 2-mL microcentrifuge tubes containing a piece of paper on which the insects gathered (Figure 1).

**Figure 2:**
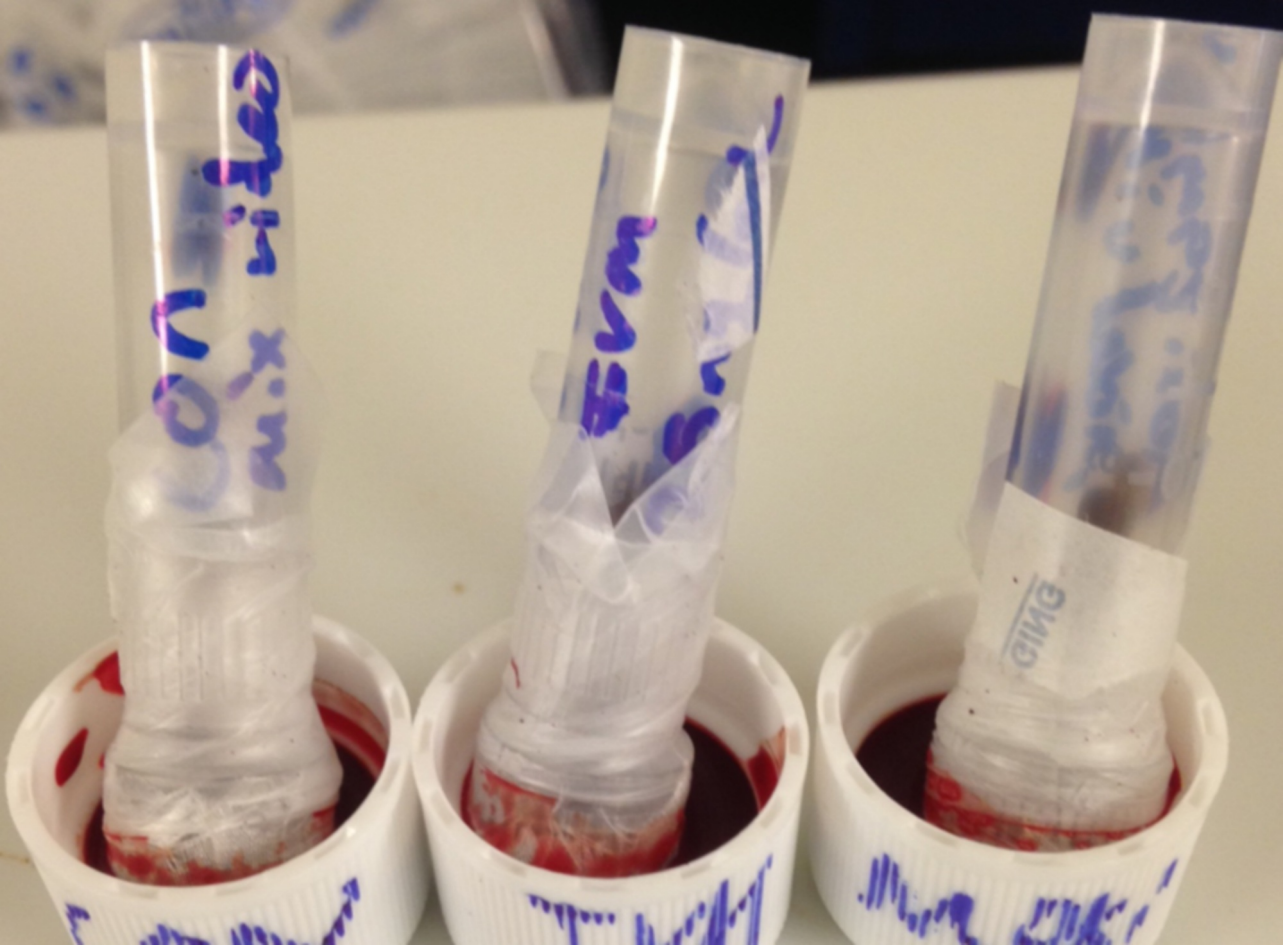
An example of bed bugs feeding on warmed blood

A 1/4-inch hole was drilled in the top of each centrifuge tube cap, and a piece of sheer fabric was glued to the surface, which was then covered with parafilm (Figure 2). Inverting this tube into warm blood allowed the bed bugs to feed (Figure 2).

**Figure 3:**
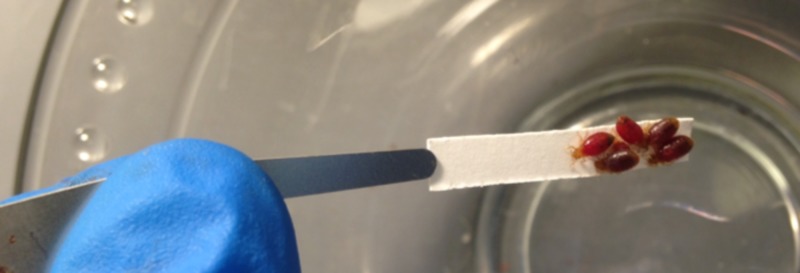
An example of fed bed bugs

Unfed bed bugs were removed and discarded. Fed *C. lectularius* were kept and observed, and all bed bugs fed only once (Figure 3).

Spinosad was purchased from Toronto Research Chemicals and diluted to a stock concentration of 2.63 mg/mL with dimethyl sulfoxide (DMSO). Fluralaner was purchased from Cayman Chemicals and diluted to a stock concentration of 10 mg/mL with DMSO. For experiment 1, the stock drug was further diluted with DMSO to the correct concentration such that 2 microliters of drug were added to 1.998 mL of defibrinated sheep’s blood (Hemostat Laboratories) to achieve the final desired concentration. For controls, 2 microliters of DMSO was added to 1.998 mL of defibrinated sheep’s blood. Observations were made after the bed bug feeding on day 0 and incapacitation rates were recorded on days 7 and 22 post-feeding.

DMSO has previously been reported to have bed bug toxicity [12]. To minimize DMSO toxicity, experiments 2 and 3 were performed with 2 microliters of the stock drugs diluted with Hanks’s Buffered Salt Solution (HBBS) to a concentration such that when 10 mcL of the diluted drug was added to 1.990 mL of defibrinated sheep’s blood the final drug concentration was achieved. For controls, 10 microliters of DMSO was diluted 1:5,000 in HBSS and added to 1.990 mL of defibrinated sheep’s blood. Bed bug incapacitation rates were recorded on days four and 14 for experiment 2 and on days two and 14 for experiment 3. Bed bugs were recorded as being either an adult or nymph throughout experiment 2. The life stages for each insect used in experiment 3 were not recorded. All experiments had two control groups, which were combined for the analysis.

The primary endpoint was bed bug incapacitation, which was defined as insects that were dead, largely immobile, or unable to remain attached to the paper perch inside the tube. The incapacitation rate (No. of incapacitated insects/No. of fed insects in the group) for each drug dose was compared with the incapacitation rate in the control group for each time point. Differences in the incapacitation rates of spinosad and fluralaner were compared to the corresponding incapacitation rates in the control group and assessed for statistical significance using a comparison of proportions calculator (https://www.medcalc.org/calc/comparison_of_proportions.php). Statistical significance was set at *P* < 0.05.

## Results

There were a total of 917 fed *C. lectularius* used in the three experiments including 387 for experiment 1, 207 for experiment 2, and 413 for experiment 3. The percentage of nymphs in each feeding group in experiment 1 ranged between 81% and 96% and 81% and 96% for experiment 2.

Experiment 1

The number and life stage for all fed *C. lectularius* are listed in Table 1. All bed bugs, with the exception of the controls and fluralaner 10 ng/mL, were incapacitated within hours of the feeding (Table 1).

**Table 1:** Life stages of C. lectularius used in experiment 1 *All incapacitated within hours of feeding

	Male	Female	5^th^ instar	4^th^ instar	3^rd^ instar	2^nd^ instar	1^st^ instar	Total number of fed C. lectularius	% nymphs in fed population
Control	8	3	2	10	26	27	7	83	87
Spinosad 5,000 ng/mL*	4	0	1	7	8	3	1	24	83
Spinosad 1,000 ng/mL*	3	3	2	4	10	5	5	32	81
Spinosad 500 ng/mL*	2	1	3	5	12	10	15	48	94
Spinosad 100 ng/mL*	1	2	5	5	6	4	3	26	88
Fluralaner 5,000 ng/mL*	3	3	2	1	6	7	9	31	81
Fluralaner 1,000 ng/mL*	5	2	2	4	6	6	15	40	83
Fluralaner 500 ng/mL*	1	1	2	6	10	8	19	47	96
Fluralaner 100 ng/mL*	4	3	3	5	9	6	9	39	82
Fluralaner 10 ng/mL	6	0	0	1	4	3	3	17	65

On day 7 post-feeding, the incapacitation rate was 28% in the control group, 58% in the spinosad 100 ng/mL group, and 100% in the spinosad 500, 1,000, and 5,000 ng/mL groups (Table 2). It was 100% in the fluralaner at 10, 100, 500, 1,000, and 5,000 ng/mL groups (Table 2). All doses were significantly different than those from the control group (p < 0.01; Table 2). Some insects were able to advance their life stage over the course of the week (e.g. 5th instar controls).

**Table 2:** Incapacitation rates day 7 post-feeding (number of healthy C. lectularius at that life stage/number of fed C. lectularius on day 0) for experiment 1 *indicates that over 7 days more 4th instars became 5th instars than were originally fed 5th instars

	Adult	5^th^ instar	4^th^ instar	3^rd^ instar	2^nd^ instar	1^st^ instar	Total incapacitation
Control	11/11	7/2*	6/10	20/26	9/27	7/7	28%
Spinosad 5,000 ng/mL	0/4	0/1	0/7	0/8	0/3	0/1	100% (p < 0.001)
Spinosad 1,000 ng/mL	0/6	0/2	0/4	0/10	0/5	0/5	100% (p < 0.001)
Spinosad 500 ng/mL	0/3	0/3	0/5	0/12	0/10	0/15	100% (p < 0.001)
Spinosad 100 ng/mL	3/3	1/5	4/5	2/6	0/4	1/3	58% (P = 0.005)
Fluralaner 5,000 ng/mL	0/6	0/2	0/1	0/6	0/7	0/9	100% (p < 0.001)
Fluralaner 1,000 ng/mL	0/7	0/2	0/4	0/6	0/6	0/15	100% (p < 0.001)
Fluralaner 500 ng/mL	0/2	0/2	0/6	0/10	0/8	0/19	100% (p < 0.001)
Fluralaner 100 ng/mL	0/7	0/3	0/5	0/9	0/6	0/9	100% (p < 0.001)
Fluralaner 10 ng/mL	0/6	0/0	0/1	0/4	0/3	0/3	100% (p < 0.001)

On day 22 post-feeding, the incapacitation rate was 61% in the control group, 88% in the spinosad 100 ng/mL group, and 100% in the spinosad 500, 1,000, and 5,000 ng/mL groups (Table 3). It was 100% in the fluralaner 10, 100, 500, 1,000, and 5,000 ng/mL groups (Table 3). All doses were significantly different than the control (p < 0.05) (Table 3). The high mortality rates in the control group were likely due to DMSO toxicity, and even with high mortality in the control groups, bed bug mortality rates in the spinosad and fluralaner groups were all significantly higher.

**Table 3:** Incapacitation rates 22 days post-feeding (number of healthy C. lectularius at that life stage/number of fed C. lectularius on day 0) for experiment 1

	Adults	Adult incapacitation rate	Nymphs	Nymph incapacitation rate	Eggs	Total incapacitation
Control	5/11	29%	8/72	69%	0	61%
Spinosad 5,000 ng/mL	0/4	100%	0/20	100%	0	100% (p < 0.001)
Spinosad 1,000 ng/mL	0/6	100%	0/26	100%	0	100% (p < 0.001)
Spinosad 500 ng/mL	0/3	100%	0/45	100%	0	100% (p < 0.001)
Spinosad 100 ng/mL	3/3	0%	0/23	100%	0	88% (P = 0.03)
Fluralaner 5,000 ng/mL	0/6	100%	0/25	100%	0	100% (p < 0.001)
Fluralaner 1,000 ng/mL	0/7	100%	0/33	100%	0	100% (p < 0.001)
Fluralaner 500 ng/mL	0/2	100%	0/45	100%	0	100% (p < 0.001)
Fluralaner 100 ng/mL	0/7	100%	0/32	100%	0	100% (p < 0.001)
Fluralaner 10 ng/mL	0/6	100%	0/11	100%	0	100% (p < 0.001)

Experiment 2

Table 4 shows the number of fed *C. lectularius* and whether they were adults or nymphs.

**Table 4:** Life stages of fed C. lectularius used in experiment 2

	Adults	Nymphs	Total number of fed C. lectularius	% Nymphs in fed population
Control	15	42	57	74
Spinosad 100 ng/mL	7	18	25	72
Spinosad 10 ng/mL	5	29	34	85
Fluralaner 10 ng/mL	4	29	35	83
Fluralaner 1 ng/mL	8	16	25	64
Fluralaner 0.1 ng/mL	8	23	31	74

Four days post-feeding, the incapacitation rate for spinosad 100 ng/mL was 8%, spinosad 10 ng/mL was 18%, fluralaner 10 ng/mL was 9%, fluralaner 1 ng/mL was 0%, and fluralaner 0.1 ng/mL was 10% which were all not significantly different (*p* > 0.05) than the control group which was 9% (Table 5). Some insects were able to advance their life stage after the feeding and before incapacitation was determined (e.g. fluralaner 1 ng/mL exposed adults).

**Table 5:** Incapacitation rate for fed bed bugs in experiment 2, four days post-feeding (number of healthy C. lectularius/number of originally fed C. lectularius on day 0) *indicates that there was one more insect categorized as an adult, rather than a nymph, 4 days post-feeding

	Incapacitation rate for Adults	Incapacitation rate for nymphs	Total incapacitation rate
Control	11/15	41/42	9%
Spinosad 100 ng/mL	8/7*	15/18	8% (p = 0.88)
Spinosad 10 ng/mL	4/5	24/29	18% (p = 0.21)
fluralaner 10 ng/mL	3/4	29/29	9% (p = 1.0)
fluralaner 1 ng/mL	9/8	16/16	0% (p = 0.12)
fluralaner 0.1 ng/mL	8/8	20/23	10% (p = 0.88)

Fourteen days post-feeding, the incapacitation rate for spinosad 100 ng/mL was 20%, spinosad 10 ng/mL was 38%, fluralaner 10 ng/mL was 17%, fluralaner 1 ng/mL was 20%, and fluralaner 0.1 ng/mL was 29% which were all not significantly different (p > 0.05) than the control group which was 28% (Table 6). Eggs and 1st instars were present in all treatment groups (Table 6).

**Table 6:** Life stages of fed bed bugs used in experiment 2, 14 days post-feeding (number of healthy C. lectularius/number of originally fed C. lectularius on day 0)

	Incapacitation rate for Adults	Incapacitation rate for Nymphs	Total incapacitation rate	Total number of eggs laid	New 1^st^ instars joining population
Control	10/15	31/42	28%	16	9
Spinosad 100 ng/mL	8/7	12/18	20% (p = 0.44)	2	2
Spinosad 10 ng/mL	4/5	17/29	38% (p = 0.32)	7	3
Fluralaner 10 ng/mL	3/4	26/29	17% (p = 0.23)	1	1
Fluralaner 1 ng/mL	8/8	12/16	20% (p = 0.45)	8	4
Fluralaner 0.1 ng/mL	8/8	14/23	29% (p = 0.92)	8	4

Experiment 3

On day 2, the post-feeding incapacitation rate was 18% in the spinosad 100 ng/mL group, 56% in the spinosad 500 ng/mL group, 78% in the spinosad 1,000 ng/mL group, and 100% in the spinosad 5,000 ng/mL group which were all significantly different than the incapacitation rate of 3% in the control group (*p* < 0.05 for all; Table 7). The incapacitation rate for fluralaner was 61% in the 100 ng/mL group and 100% for fluralaner 500, 1,000, and 5,000 ng/mL groups which were significantly different from the control (*p* < 0.001 for all; Table 7). On day 14, the post-feeding incapacitation rate was 13% in the spinosad 100 ng/mL group, 44% in the spinosad 500 ng/mL group, 76% in the spinosad 1,000 ng/mL group, and 100% in the spinosad 5,000 ng/mL group. The incapacitation rates for spinosad 500 ng/mL, 1,000 ng/mL, and 5,000 ng/mL were all significantly different from the control group, respectively (*p* < 0.05 for all; Table 7). The incapacitation rate for fluralaner was 49% in the 100 ng/mL group and 100% for fluralaner 500, 1,000, and 5,000 ng/mL groups which were significantly different than the control (*p* < 0.001 for all; Table 7).

**Table 7:** Incapacitation rates for fed bed bugs used in experiment 3 on days 2 and 14 post-feeding

	Number of fed C. lectularius	Day 2 incapacitation rate	Day 14 incapacitation rate
Control	90	3%	14%
Spinosad 5,000 ng/mL	37	100% (p < 0.001)	100% (p < 0.001)
Spinosad 1,000 ng/mL	45	78% (p < 0.001)	76% (p < 0.001)
Spinosad 500 ng/mL	39	56% (p < 0.001)	44% (p < 0.001)
Spinosad 100 ng/mL	38	18% (p = 0.003)	13% (p = 0.88)
Fluralaner 5,000 ng/mL	28	100% (p < 0.001)	100% (p < 0.001)
Fluralaner 1,000 ng/mL	35	100% (p < 0.001)	100% (p < 0.001)
Fluralaner 500 ng/mL	50	100% (p < 0.001)	100% (p < 0.001)
Fluralaner 100 ng/mL	51	61% (p < 0.001)	49% (p < 0.001)

**Figure 4:**
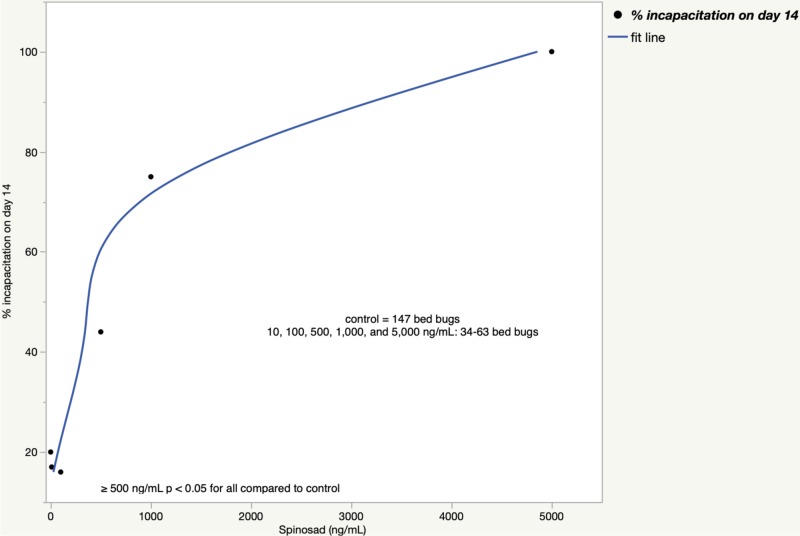
Bed bug incapacitation rate 14 days after a spinosad blood meal (experiments 2+3)

**Figure 5:**
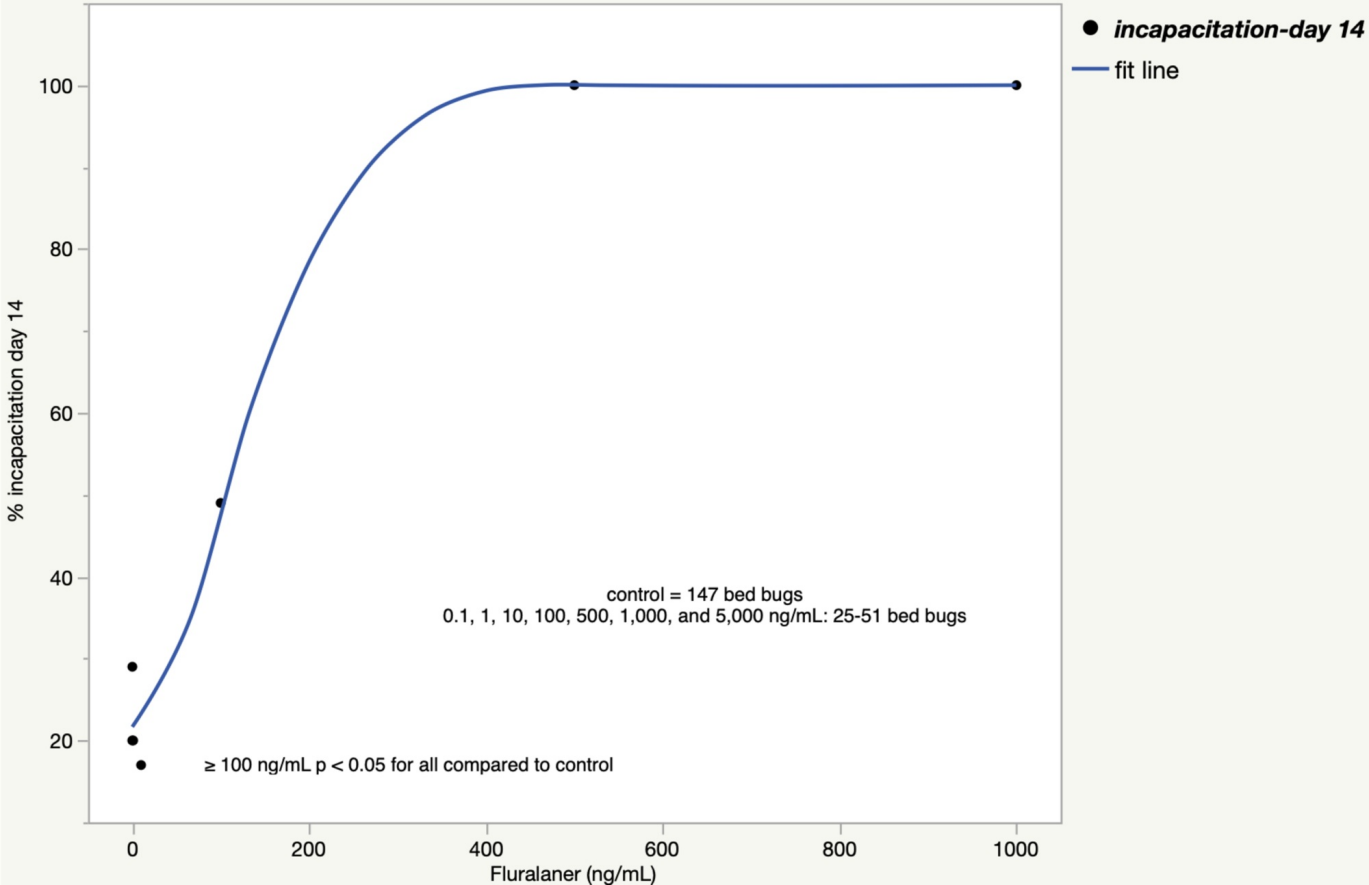
Bed bug incapacitation rate 14 days after a fluralaner blood meal (experiments 2+3)

The incapacitation rates when experiments 2 and 3 were combined are shown in Figure 4 for spinosad and Figure 5 for fluralaner.

## Discussion

Both spinosad and fluralaner showed a dose-response effect on *C. lectularius* incapacitation. At ≥500 ng/mL, fluralaner incapacitated all fed bed bugs. The results are preliminary and additional studies are needed to further explore the toxic effects these drugs have on bed bugs. Future studies could include administering the drugs to animals, measuring the plasma concentrations of the drugs in the animals, and simultaneously observing the *C. lectularius* morbidity and mortality over time for each life stage in the fed insects.

If spinosad was ever to be used as an oral chemotherapeutic agent in humans, and the pharmacokinetics are similar in humans as in dogs, then the plasma concentration of spinosad would be less than 500 ng/mL within 1 day [18]. If spinosad was ever to be used by humans to help control a bed bug infestation, it would likely mean that multiple doses of the drug would be required.

Fluralaner has a much longer plasma half-life than spinosad [17-18,20-22]. A single dose of fluralaner in dogs resulted in plasma concentrations greater than 500 ng/mL for more than 21 days [22]. If the pharmacokinetics of fluralaner in dogs are similar in humans, this could mean that a person could take a single oral dose of fluralaner and would have over two weeks of plasma concentrations that achieved 100% bed bug incapacitation in our experiments which could be sufficient time for all bed bugs in a population to take a toxic blood meal [22].

Fluralaner also rapidly kills *Phlebotomus* sand flies and *Anopheles*, *Culex*, and *Aedes* mosquitoes [23-25]. Preclinical modeling estimates that one oral 410 mg dose of fluralaner in humans would provide 50 to 90 days of insecticidal effect against Phlebotomus sand flies and mosquitoes [25]. However, there have been no human clinical trials of oral fluralaner or spinosad reported in clinicaltrials.gov or published in PUBMED.gov. There is only one case report of an intentional overdose of spinosad [26]. If fluralaner is ever shown to be safe, in humans it could then be further evaluated for its ability to harm *C. lectularius.*

Limitations

The number and life stages for the *C. lectularius* were not held constant at each of the tested doses. Additionally, the time from the laboratory feeding until the incapacitation rates were calculated was not constant. The experiments were not set up to examine fecundity, the ability of the insects to refeed, molt, or ambulate. Experiment 1, and to a lesser extent, experiments 2 and 3, had high mortality in the control group which is hypothesized to be secondary to DMSO. Differences between pyrethroid-sensitive and resistant bed bugs, as well as recently field-captured versus laboratory-raised insects, were not assessed.

The experimental design did not account for any drug metabolism or intracellular accumulation, which would otherwise occur *in vivo*, and so incapacitation rates for bed bugs fed on humans or animals, given spinosad or fluralaner, might be different than what was found in our laboratory feedings. Additionally, the doses that were used in the laboratory feedings were based on the plasma levels of the drugs recorded in animals given the drugs at therapeutic doses. However, hematophagous insects take whole blood meals and not plasma meals, and so the actual drug concentration obtained from a feeding insect would likely be different than what would be reported as the plasma concentration.

## Conclusions

Preliminary results show that both spinosad and fluralaner are able to incapacitate *C. lectularius* in a dose-responsive manner. Doses of ≥500 ng/mL of fluralaner in spiked blood meals were able to incapacitate all *C. lectularius*. Doses of ≥1,000 ng/mL of spinosad in spiked blood meals were able to incapacitate a majority of *C. lectularius*. If either drug, but especially fluralaner due to its favorable pharmacokinetics and safety profile in animals, is approved for human use, then further studies should be undertaken to evaluate their ability to eliminate bed bug infestations.
